# Tackling Regional Public Health Issues Using Mobile Health Technology: Event Report of an mHealth Hackathon in Thailand

**DOI:** 10.2196/mhealth.8259

**Published:** 2017-10-16

**Authors:** Atipong Pathanasethpong, Chitsutha Soomlek, Katharine Morley, Michael Morley, Pattarawit Polpinit, Alon Dagan, James W Weis, Leo Anthony Celi

**Affiliations:** ^1^ Department of Anesthesiology Faculty of Medicine Khon Kaen University Khon Kaen Thailand; ^2^ Department of Computer Science Faculty of Science Khon Kaen University Khon Kaen Thailand; ^3^ Harvard Medical School Boston, MA United States; ^4^ Department of Computer Engineering Faculty of Engineering Khon Kaen University Khon Kaen Thailand; ^5^ Department of Emergency Medicine Beth Israel Deaconess Medical Center Boston, MA United States; ^6^ Massachusetts Institute of Technology Cambridge, MA United States

**Keywords:** hackathon, mHealth, interdisciplinary collaboration

## Abstract

Hackathons are intense, short, collaborative events focusing on solving real world problems through interdisciplinary teams. This is a report of the mHealth hackathon hosted by Khon Kaen University in collaboration with MIT Sana and faculty members from Harvard Medical School with the aim to improve health care delivery in the Northeast region of Thailand. Key health challenges, such as improving population health literacy, tracking disease trajectory and outcomes among rural communities, and supporting the workflow of overburdened frontline providers, were addressed using mHealth. Many modifications from the usual format of hackathon were made to tailor the event to the local context and culture, such as the process of recruiting participants and how teams were matched and formed. These modifications serve as good learning points for hosting future hackathons. There are also many lessons learned about how to achieve a fruitful collaboration despite cultural barriers, how to best provide mentorship to the participants, how to instill in the participants a sense of mission, and how to match the participants in a fair and efficient manner. This event showcases how interdisciplinary collaboration can produce results that are unattainable by any discipline alone and demonstrates that innovations are the fruits of collective wisdom of people from different fields of expertise who work together toward the same goals.

## Introduction

### Khon Kaen University and its Role in the Health Care of Northeast Thailand

Health care for the population of Thailand is covered under a universal coverage scheme subsidized by the government [1]. Every citizen is entitled to free health care accessed by first visiting a local primary health center. Patients requiring more advanced capabilities are referred to more sophisticated facilities. In this system, university-based health centers serve as the pinnacle of advanced care and are the ultimate referral destinations for patients with complex diseases and conditions.

Khon Kaen University (KKU) serves as the apex of health care and advanced training in health sciences for northeast Thailand. As there is a diverse array of health problems specific to each region, KKU is positioned at the frontline of research on issues unique to the northeast, such as melioidosis [2] and cholangiocarcinoma [3]. In addition, KKU also is an emerging leader in education in health and computer technology. The university offers advanced training in both areas. The Health Science Faculties comprises the Faculty of Medicine, Faculty of Dentistry, Faculty of Nursing, Faculty of Pharmaceutical Sciences, Faculty of Associated Medical Sciences, Faculty of Veterinary Medicine, and Faculty of Public Health. Training in computer technology is led by the Faculty of Engineering and Faculty of Science, with the former focusing more on hardware and the latter focusing more on software.

Building research and innovation via interdisciplinary collaboration is a key element of the mission of KKU. Despite the encouragement of the university leadership, there have not been many significant successful collaborations around health care and technology. One of the barriers was finding the opportunity to bring together individuals from the different disciplines to address key health challenges, such as improving population health literacy, tracking disease trajectory and outcomes among rural communities, and supporting the workflow of overburdened frontline providers. The prospect of developing and implementing a health care hackathon created a valuable opportunity to achieve this goal. In addition to providing this opportunity, the Harvard–Massachusetts Institute of Technology (MIT) Division of Health Science and Technology (HST) Sana team provided the experience and knowledge to conduct a hackathon, opening the door to new innovations and collaboration.

### MIT Sana and Hackathons

Hackathons are intense, short, collaborative events focused on creating innovative solutions for pressing problems [4,5]. Over the last 3 years, the Laboratory for Computational Physiology (LCP) at HST, which hosts Sana, has organized more than a dozen of these events to imagine new ways technology can address critical health care challenges and develop local capacity that is fully supported by global networks.

Sana is a global consortium of academic partners with an interest in leveraging mobile health technology (mHealth) to improve health care delivery in resource-constrained settings. The hackathons have brought together students and professionals from different disciplines such as engineering, computer science, medicine, social service, public health, and business to design and develop mHealth applications.

**Table 1:** Health hackathons organized by the Massachusetts Institute of Technology Laboratory for Computational Physiology.

Date		Event	Location
**2017**			
	July	Health Data Workshops	Cebu, Philippines
	July	Health Datathon	Singapore
	June	Mobile Health Hackathon	Mexico City, Mexico
	May	Health Datathon	Sao Paulo, Brazil
	April	Hacking Discrimination Hackathon	MIT^a^
	March	Health Datathon	Melbourne, Australia
	January	Mobile Health Hackathon	Khon Kaen, Thailand
**2016**			
	December	Health Datathon	London, United Kingdom
	October	Health Datathon	Beijing, China
	September	Internet of Things Hackathon	Taipei, Taiwan
	August	Hacking Mobile Health Hackathons	MIT
	January	Mobile Health Hackathon	Mexico City, Mexico
**2015**			
	October	Mobile Health Hackathon	Thessaloniki, Greece
	September	Health Datathon	MIT and London, United Kingdom
	July	Mobile Health Hackathon	Kampala, Uganda
	June	Mobile Health Hackathon	Popayan, Colombia
**2014**			
	September	Health Datathon	MIT, London, United Kingdom, and Paris, France
	January	Health Datathon	MIT

^a^MIT: Massachusetts Institute of Technology.

## Methods

### Implementation of Khon Kaen University mHealth Hackathon

As an academic institution, KKU decided that the event should benefit both society and the university’s internal stakeholders (university missions, faculty members, and students). With this guiding principle, the goals of the event were as follows:

- Solve public health issues in the northeast and Thailand using mHealth
- Promote collaborations between faculty members
- Provide participants with a learning opportunity that is hands-on and interdisciplinary
- Provide participants with international exposure and collaboration
- Foster research and innovation

The organizational process and structure of the event addressed these goals, along with incorporating modifications to recognize Thai culture.

### Team Size and Composition

The KKU hackathon differed significantly from other hackathons in the participant recruitment and selection process. Instead of having participants joining individually, the organizing committee decided to form teams from health sciences and computer technology ahead of the event and match and merge the teams at the beginning of the event. Because assertiveness and outspokenness are not the norm for Thai people [6], this approach was aimed at reducing the stress of getting acquainted within a short amount of time and forming impromptu teams.

As the optimal team size for software development is suggested to be 7 individuals [7], the organizers decided that each team would have 2 to 3 members from health care, 2 members from computer technology, and 2 members from other related disciplines such as design and business.

### Recruiting Health Science Teams

Each of the Health Science Faculties was allocated a number of teams based on its size and readiness. Each faculty was tasked with recruiting teams with the conditions that each team bring with it a specific and distinct health problem to be solved with mHealth technology and at least one health professional from the team be continually present during hack days.

Selection of teams was done internally by the leadership of each faculty, as it was felt they knew best about its disciplines and internal characteristics. The organizing committee accepted all teams that had been selected by their faculties.

### Recruiting Computer Technology Teams

The Department of Computer Science and Department of Computer Engineering announced recruitment for the event in their respective departments. Initially, 35 students from computer science and 53 students from computer engineering were interested in joining the hackathon. Interested students progressed through a series of training activities that would prepare them for the event. Students who regularly attended the activities were moved to the top of the candidate list. Finally, candidates answered a short online exam and underwent an interview process. Based on student academic records, training, and the interview, the Department of Computer Science, Faculty of Science, and the Department of Computer Engineering, Faculty of Engineering, each selected 20 candidates.

The 40 candidates were then merged into 20 teams, each with one computer science student and one computer engineering student. Each pair was allowed to recruit 2 additional members from other related disciplines to complete a team of 4 members.

### Matching the Teams from Health Care and Computer Technology

The organizing committee felt it was essential that the health care and engineering teams have the opportunity to find their best match, given that each project and team had specific needs and capabilities. In order to accomplish this, the organizing committee came up with an innovative approach: using a speed-dating format [8] for team introduction followed by using the US National Resident Matching Program algorithm [9] to match teams. The main goals were to allow the teams to get acquainted in a short time and match them efficiently and fairly.

To mimic the speed-dating formatting, we set up a circle of 20 tables and asked each health care team to stay inside the circle and remain stationary at its assigned table. The computer technology teams lined up outside the circles. The teams had 3 minutes to talk to each other and 30 seconds to reflect on the conversation. When the time was up, each computer technology team moved to the next table and the process began anew until every team had met all teams from the other group. Each team then ranked up to 10 teams from the other group based on its levels of preference. The rankings from all 40 teams were collected, and 2 members of the organizing committee manually applied the algorithm to match the teams. A total of 18 pairs were matched with the algorithm, and 2 pairs that could not be matched were matched with a coin toss.

## Results

### Bootcamp

Prior to the actual hackathon, a 2-day bootcamp was conducted. The purpose of the bootcamp was to introduce key concepts on designing mHealth innovations and methods of collaboration between clinicians and engineers, as well as address how to implement and evaluate mHealth solutions.

To provide content to a diverse audience, general topics relevant to all participants were covered in the mornings. In the afternoons, a variety of workshops was provided for participants to choose from, each addressing specific skills and/or subject matters. All lectures were given in English, with opportunities to ask questions in Thai. The language of each workshop was determined by its instructors.

### Hack Days

Participants were given 48 hours of hack time to complete their projects and submit their presentation. It was not required to have a fully developed, functioning app by presentation day. Instead, the requirements were for the teams to design products that would showcase their ideas and also show that a minimum viable product is feasible.

All teams were housed in the main hall of the KKU central library, with each having its own working station, network sockets, and power outlets. Participants brought their own electronic equipment. There was a team from KKU and mentors to help with miscellaneous requests from the participants. Refreshments were provided throughout the event.

There were both Thai and international mentors. In total, there were 4 mentors from MIT, 2 from Harvard Medical School (HMS), 1 from University of Waterloo, and 16 from KKU. Mentoring was provided throughout the hackathon and during scheduled mentor rounds. There was a board on which participants could write their problems. Mentors would periodically check the board and offer help if the posted problems were within their fields of expertise.

There were also mentor rounds and meetings throughout the event. During rounds, groups of mentors roamed the event hall to check the teams’ progress and provide answers to problems. In addition, each team also had 2 scheduled mentor meetings in a private room, during which it reported its progress to a panel of mentors.

**Table 2:** Schedule of bootcamp and hack days.

Date	Time	Activity
January 9, 2017	9:00-9:30 AM	Introduction and opening ceremony
	9:30-10:15 AM	Solving the Problems of Health Care
	10:30-11:15 AM	Design Thinking in Global Health Informatics
	11:15 AM-12:00 PM	Bridging the Divide Between Information Technology Developers and Clinicians
	1:00-1:45 PM	Asia eHealth Information Network
	1:45-3:15 PM	Workshops in breakout rooms
January 10, 2017	9:00 -9:45 AM	The Formula for Good Health
	9:45-10:30 AM	Bringing eHealth Solutions to Market
	11:00 AM-12:00 PM	Creating a Culture of Entrepreneurship
	1:00-2:30 PM	Workshops in breakout rooms
	2:30-4:00 PM	Team introduction and matching
January 11, 2017	8:30 AM onward	Full day of hackathon
January 12, 2017		Full day of hackathon
January 13, 2017	8:30 AM	Deadline for submitting presentations
	8:40-11:20 AM	Team presentation
	11:20-11:40 AM	Deliberation of winners
	11:40 AM-12:00 PM	Award ceremonies and closing of event

### Judging

There were 3 judges from the MIT Sana team, 2 judges from KKU, and 2 judges from Thai public health organizations. The main judging criteria were innovativeness, feasibility, and value of the projects. The winning team created a mobile app to optimize the logistics of moving patients around in a hospital. The first runner-up built a mobile app to incentivize blood donation and make the process more convenient for donors. The second runner-up tackled a mobile app that would allow patients on continuous peritoneal dialysis to collect data and better communicate with their caregivers.

### Handling of Intellectual Properties

All participants ceded their rights over the creations to KKU under an agreement. With this agreement, KKU would be the sole entity overseeing commercialization of the products, with potential revenues to be shared between KKU and the teams. Having KKU as the sole owner of intellectual property would facilitate administrative processes regarding the projects.

One of the goals was to benefit the public as much as possible. To strike the right balance, KKU released all ideas and products from the event to the public under Creative Commons License Attribution-NonCommercial 4.0 International [10]. With this license, the public would be able to further develop the ideas and products, as long as such ventures are noncommercial.

## Discussion

### Building a Fruitful Collaboration

The interest, hands-on engagement, and strong support of the leadership in each of the academic departments and administration at KKU were key factors contributing to the success of the KKU mHealth hackathon. From the beginning, this level of support allowed the KKU faculty members to have the time and resources necessary for planning and organizing the event, along with building enthusiasm and participation from the entire university.

There were other important elements that supported the hackathon along with laying the foundation for ongoing collaboration between KKU and the HMS-MIT teams. These included commitment, flexibility, respect for culture and local practices, and willingness to understand each other’s needs. For example, the KKU members needed to learn about the details of organizing and conducting a health care hackathon, while the HMS-MIT Sana members needed to learn about Thai health care and educational systems.

Detailed planning and careful implementation were 2 other critical elements of the successful hackathon. Use of Web-based document-sharing and Internet conference calling were critical for communication. Three KKU faculty members also had the benefit of attending an MIT Sana hackathon in Taipei, Taiwan, 4 months prior to the KKU hackathon.

### Mentoring for Success

One strength of this event compared to prior hackathons was the relatively large number of mentors. With staff members from multiple departments at KKU and a large contingent of visiting mentors from the HMS-MIT Sana team, we were able to provide a diverse and large group of clinicians, engineers, and social and data scientists as mentors. There were 25 mentors in total for 20 hackathon teams.

While the diverse perspectives were certainly useful, the increasing numbers provided new challenges. We structured the mentors into small groups comprised of both Thai and international mentors. These small groups roamed the event space answering questions and reviewing the progress of the teams in order to help ensure that specific questions were answered in a timely fashion. Teams submitted questions on a request board and mentors with the appropriate expertise responded to the teams.

As the event continued, we realized that this large group of mentors required a more structured mechanism for tracking suggestions and feedback. We wanted to strike an appropriate balance between oversight and allowing teams the space to work on their own. To this end we trialed the use of a paper log at each group’s table so that mentoring teams would be able to record basic notes on their interactions with a project group. This enabled the next mentoring team to identify whether issues had already been addressed and whether the team had recently received (potentially conflicting) advice from other mentors.

Unfortunately, given the impromptu addition of this system, it was not adopted across all mentoring groups. We look forward to implementing a better system of tracking the mentor/team interactions in the future and have begun brainstorming digital solutions that might be less cumbersome than paper logs.

### Cultural Awareness

Unlike most hackathons where participants form teams at the beginning of the event without much of a system, we decided to be more structured with team composition. An open format could be intimidating and disorienting to inexperienced participants, and it might lead to groups without a good mix of expertise and skills. Considering that the hackathon is a novel concept for Thailand, we believe that this decision was a correct one and that it was crucial to the success of the event.

### A Sense of Mission

Each team tackled a unique health issue—no 2 teams worked on the same problem. It was also required that at least 1 health care professional be present at all times during hack days to provide prompt feedback to the computer technology team. We believe that these 2 elements greatly contributed to the event, as they made the teams feel invested in their projects.

### Efficient and Fair Matching

We also believe that using the speed-dating format and the US resident matching algorithm allowed for an informed, efficient, and fair matching process. Each team had a chance to learn about all teams from the other group before deciding which teams they preferred. The time it took for this process (including orientation) was only 75 minutes. And because each team was allowed to populate its own preference list before having a well-established algorithm impartially applied to it, satisfaction in the process was high.

### Conclusion

Innovations in every discipline are the product of people coming together, typically with different perspectives but shared goals. Great accomplishments are seldom the work of a single individual, and yet we still focus on individual learning in schools, at the workplace, and in life. The hackathon provided a platform for the participants to teach and learn from each other. Although mentors provided the episodic one-to-many model of teaching, we observed continuous many-to-many model of learning throughout the event. We witnessed firsthand how a sense of mission and a feeling of trust within each group were critical to its success during the hackathon. None of us is as smart as all of us.

## Abbreviations

HMS: Harvard Medical School
HST: Health Sciences Technology
KKU: Khon Kaen University
LCP: Laboratory for Computational Physiology
MIT: Massachusetts Institute of Technology
